# Lessons from COVID-19 in South Africa: Keeping our environment clean should be the first law of health

**DOI:** 10.4102/safp.v63i1.5297

**Published:** 2021-06-15

**Authors:** Sarentha Chetty, Varsha Bangalee

**Affiliations:** 1 Department of Pharmacy and Pharmacology, Faculty of Health Sciences, University of the Witwatersrand, Johannesburg, South Africa; 2 Department of Pharmacy, Faculty of Health Sciences, University of KwaZulu-Natal, Durban, South Africa; 3 Discipline of Pharmaceutical Sciences, School of Health Sciences, University of KwaZulu-Natal, Durban, South Africa

**Keywords:** COVID-19, litter, hygiene, infection prevention, infectious disease

## Abstract

In the wake of the coronavirus disease 2019 (COVID-19) pandemic, the links between poor hygiene, unclean environments and human health cannot be overemphasised, particularly in South Africa with its high incidence of infectious diseases and overburdened health system. One very controllable factor that is often overlooked is the poor disposal of litter and waste management and its adverse effects on public health. By wearing masks, regular handwashing and sanitising, as well as making sure that neighbourhoods and public spaces are clean and safe, the spread of COVID-19 and other diseases can be prevented.

## Introduction

The link between poor hygiene and unclean environments on human health is not new.^1,2^ In 1989, the European Charter on Environment and Health, stated that: ‘Good health and well-being require a clean and harmonious environment in which physical, physiological, social and aesthetic factors are all given their due importance’ (p. 2).^3^ Amongst the environmental contributors to poor health, the impact of litter and illegal dumping has far-reaching consequences. Litter is not only unsightly and odorous, but it can also adversely impact the environment, society and the economy. Regarding public health, it serves as a breeding ground for disease vectors including mosquitoes, flies, cockroaches, rats and parasites.^4,5,6,7^ The transmission of bacterial, viral and parasitic infections through solid waste contamination of the environment is well documented leading to a host of infectious and contagious diseases.^5,6,7^ Recently the link between unclean environments and disease transmission has been revisited in light of the novel Coronavirus disease 2019 (COVID-19). The rapid global spread of COVID-19 quickly catapulted the virus to pandemic status, making it a global health emergency.^8,9^

As the world cooperatively began to learn about the virus, research increasingly emerged, regarding not just the pathology but the molecular biology, transmission and lifespan of the virus. It was confirmed that COVID-19 is spread by the inhalation of infected droplets and contact with infected surfaces^9^ or possibly through aerosol spread.^10^ Furthermore, the virus could remain transmissible from objects including metal, glass, plastic and paper for an estimated period of up to 9 days.^11^ This was a game-changing discovery that served as a reminder of the effect of environmental hygiene on our health.

Maintaining a clean environment is particularly important in South Africa, where a large population of lower socio-economic individuals and families reside in densely populated informal settlements.^12^ The advent of COVID-19 and measures to try and contain the spread have brought to light the inadequate provision and inherent weaknesses in access to clean water, sanitation and waste removal in various communities across the country,^13,14^ which collectively reinforce the risk of disease transmission.^14,15^ For the greater part, these individuals are unable to reduce social contact as they do not have the luxury to work from their homes and they live in crowded spaces where lockdown regulations present a huge challenge. The notably lower levels of hygiene in these areas, owing to poor access to clean water, present a major obstacle to overcome the pandemic in South Africa.^13,14,16^ Despite these obstacles being beyond the control of those individuals living in informal settlements, and largely the mandate of the municipality, one controllable yet underemphasised factor for all South Africans especially in public spaces, is the responsible disposal of litter. This is a simple task that can save countless lives. In addition to this, it is imperative that government ensures that appropriate waste receptacles are accessible and that basic service delivery, that is, the timeous collection of rubbish and cleaning of streets and public spaces is conducted.

As the country continues to loosen its lockdown restrictions, more South Africans are returning to work and venturing out into shared public spaces. As a precautionary measure, the mandatory use of face masks, as well as increased use of hand sanitisers and other protective equipment, have been advocated. Surgical masks are highly susceptible to being exposed to the virus because of respiratory droplets shed on them. These masks could also be infected with other pathogens, for example, meningitis or Hepatitis B. The majority of disposable masks are manufactured from plastic-based materials that are liquid-resistant and durable long after they have been discarded.^17^ Poor disposal of single-use masks, sanitisers and gloves, amongst other protective equipment, has resulted in a surge of medical wastes in the environment.^17^

This waste, together with other litter, has been finding its way to shorelines and landfills, where it is contaminating the soil and water and adding to an already polluted environment. The impact of this amount of waste is far-reaching and will affect plant, marine and animal ecosystems for years to come.^17^ The use of face masks is being advocated in a number of countries. Reports globally show that since the start of the COVID-19 pandemic there has been a huge upsurge in medical waste.^18,19^ Procedures for the safe disposal of medical waste within healthcare facilities have been suggested by the WHO,^20^ however a gap exists in domestic disposal and in disposal of contaminated waste outside healthcare facilities. Contagious waste could present an infection risk for other family members, waste collection workers and other members of the public. This could also present a risk of disease outbreaks.^18,21^ Several countries have recognised this risk and have adopted methods to mitigate the spread of the virus, which include special public bins and waste disposal procedures.^18^ This is something that South Africa should investigate and provide more public guidance.

To date, COVID-19 has claimed over 3 116 000 lives worldwide.^22^ In South Africa, a country that already has a high burden of infectious diseases, the largest number of HIV infections and the fourth largest TB population globally, this new public health crisis is bound to increase the strain on an already overburdened health system.^23,24^ COVID-19 has directly and indirectly affected the functionality of the health system. In the early days of the pandemic, efforts to curb the spread of the virus resulted in reorganising the existing health workforce, as well as the suspension of certain services. Fear of contracting the disease also resulted in a reduced number of patients seeking medical attention. The overwhelming use of the health system by COVID-19 patients can compromise the ability of the health system to deliver other routine essential medical services.^23,24^ This could also increase morbidity and mortality from diseases other than COVID-19 and further increase the cost to the health system.^23^

Despite the appearance of promising vaccines,^25^ the focus in South Africa should still be on how to mitigate the spread of infection by improving access to clean water, good sanitation and increasing public awareness of pollution and littering and personal hygiene, as these strategies will continually impact the future health of the country. In the prevailing circumstances, the importance of infection prevention cannot be overstressed. We each have a role by being vigilant in the wearing of masks, regular handwashing and sanitising. By also making sure our neighbourhoods and public spaces are clean and safe, we can influence the spread of not just COVID-19 but other diseases too.

As the lockdown regulations ease and we venture back into our pre-COVID-19 existence, it is important that we heed the lessons of good hygiene in mitigating the spread of disease. The value of good personal hygiene and a clean environment should be imprinted in our memories. In the near future, governments should encourage discussion amongst public health experts, environmental scientists and epidemiologists to highlight the effect of an unclean environment on the health of the population. If we each do our part: the public by adhering to rules regarding responsible disposal of litter and the government by providing better service delivery, socio-economic development and investment and stronger enforcement of environmental regulations, we can ensure that common spaces are both aesthetically appealing and clean and safe. As the age-old advice goes ‘Prevention is better than cure’.
